# Recent Advances in Surface Activation of Polytetrafluoroethylene (PTFE) by Gaseous Plasma Treatments

**DOI:** 10.3390/polym12102295

**Published:** 2020-10-07

**Authors:** Gregor Primc

**Affiliations:** Jozef Stefan Institute, Jamova cesta 39, 1000 Ljubljana, Slovenia; gregor.primc@ijs.si

**Keywords:** gaseous plasma, discharge parameters, fluorinated polymers, polytetrafluoroethylene, surface wettability

## Abstract

Fluorinated polymers are renowned for their chemical inertness and thus poor wettability and adhesion of various coatings. Apart from chemical methods employing somewhat toxic primers, gaseous plasma treatment is a popular method for the modification of surface properties. Different authors have used different plasmas, and the resultant surface finish spans between super-hydrophobic and super-hydrophilic character. Some authors also reported the hydrophobic recovery. The review of recent papers is presented and discussed. Correlations between plasma and/or discharge parameters and the surface finish are drawn and the most important conclusions are summarized. The concentration of oxygen in the surface film as probed by X-ray photoelectron spectroscopy is inversely dependent on the concentration of oxygen in gaseous plasma. The predominant mechanism leading to hydrophilic surface finish is bond scission by deep ultraviolet radiation rather than functionalization with reactive oxygen species.

## 1. Introduction

The surface properties of polymers depend on numerous parameters including composition, structure and morphology, as well as properties of any foreign material that might have been adsorbed. The surface properties may not always be adequate, so they have to be modified for an appropriate adhesion of various coatings, including glues and inks. A traditional method for tailoring the surface properties of polymers is the application of primers. These chemicals stick to the surface of a polymer and assure the appropriate adhesion of the desired coating. The primers are not always ecologically benign, so there is a trend of suppressing their applications. The most popular alternative is the application of gaseous plasma, in particular non-equilibrium gaseous plasma sustained in different gases or gas mixtures at different pressures. This technique works well for a range of polymers, excluding fluorinated ones. A comprehensive review of new developments in surface functionalization and the nanostructuring of fluorine-free polymers using controlled plasma treatments has been published in several papers, including [1,2,3,4,5,6,7].

Gaseous plasma is a source of charged particles (particularly free electrons and positively charged ions, sometimes also negatively charged ions), neutral reactive particles (usually molecular fragments including free atoms), and radiation. The surface finish depends on the fluxes or fluences of all plasma constituents onto the surface of the polymer. The complete characterization of gaseous plasma is not a trivial task, so most authors who have used gaseous plasma for tailoring the surface properties of polymer materials skip the influence of plasma parameters on the surface finish and prefer reporting discharge parameters. The discharge power (or power density), type of discharge, gas pressure and/or flow, as well as treatment time, are often reported. Plasma parameters depend on discharge parameters, but the dependence is not always straightforward. Many authors found large differences in plasma parameters upon a slight change of discharge parameters due to non-linear effects, and mainly due to the influence of gaseous impurities on the plasma parameters. The interaction of gaseous plasma with polymer materials has been a subject of numerous scientific studies, and the results were found useful in the optimization of industrial-size reactors [8,9,10,11].

Gaseous plasma glows, so it is a source of radiation in the visible range of wavelengths. Usually, there is also significant radiation in the invisible part of the light spectrum. Of particular importance is radiation in the ultraviolet (UV) and vacuum ultraviolet (VUV) ranges of wavelengths. Such radiation is capable of breaking bonds in the polymers, so its contribution to the polymer chemistry is by far more important than the contribution of visible radiation. Furthermore, the radiation in the invisible range (UV and VUV) may be orders of magnitude more intensive than visible radiation. The penetration depth of photons in the UV and VUV range of wavelengths increases with increasing wavelength. For example, the penetration depth of soft UV radiation useful for polymer cross-linking is in the sub-millimeter range. In contrast, the radiation at the major line of a low-pressure mercury lamp (254 nm) penetrates only a few micrometers into many polymer materials. The penetration depth of VUV radiation may be only a few 10 nm. Depending on the predominant radiation type, polymers are modified over the surface layer spanning from a few 10 nm to almost a millimeter. The influence of VUV radiation on the surface properties of a model polymer was elaborated recently in [12].

The neutral radicals will not penetrate deep into a polymer upon plasma treatment. Their kinetic temperature is usually close to room temperature (RT), so any polymer modification by neutral reactive particles is limited to the very surface unless chain reactions are triggered by the interaction between a radical and the polymer. The same applies to charged particles. Their kinetic energy depends enormously on the type of discharge adopted for plasma generation. The positively charged ions impinging polymer’s surface may have various kinetic energy anywhere between a few eV and several 1000 eV. The interaction of energetic particles with polymers causes radiation damage and thus the partial or complete destruction of the surface layer. The etching is also pronounced, either by kinetic effects (sputtering) or a combination of kinetic and chemical effects (reactive ion etching).

Gaseous plasma is preferably sustained in a selected gas of high purity. In practice, this is not always feasible. For example, a vacuum system usually contains a specific concentration of water vapor that is slowly released from the surface of any vacuum component, thus contributing to the impurities in the vacuum system. Furthermore, polymer samples themselves may serve as a source of impurities. The desorption is less pronounced at atmospheric pressure, but there are very few papers on plasma characterization revealing the concentration of H, OH, and O impurities below the detection limit. Usually, the concentration of such reactive species is high enough even in a plasma sustained in noble gases, so one should take them into account at any attempt to interpret the results of plasma-treated polymers.

Although often neglected, gaseous plasma treatment may cause residual surface stress or strain [13,14,15]. This effect usually leads to modifications of the surface morphology. For example, Bruce et al. [16] explained increased roughness upon plasma treatment by buckling instability. Polymer was proposed as a bilayer structure of the stiff plasma-modified surface and soft unmodified bulk. The mismatch in the stiffness of both layers causes wrinkles to minimize the elastic energy of both layers. Both the wavelength and the amplitude of the wrinkles were found to increase linearly with the thickness of the plasma-modified layer [16]:

(1) $$\lambda =2\pi d{\left(\frac{E_{A}(1-\upsilon_{B}^{2})}{3E_{B}(1-\upsilon_{A}^{2})}\right)}^{\frac{1}{3}},$$

where *d* is a thickness of the plasma-modified layer by etching, *E*_A_ and *E*_B_ are the elastic moduli of the plasma-modified and unmodified layer and *υ*_A_ and *υ*_B_ are Poisson’s rations of both layers. The amplitude of buckling marked with *A* is [16]:

(2) $$A=d{\left(\frac{\sigma }{-\frac{E_{A}}{4(1-\upsilon_{A}^{2})}{\left(\frac{3E_{B}(1-\upsilon_{A}^{2})}{E_{A}(1-\upsilon_{B}^{2})}\right)}^{\frac{2}{3}}}-1\right)}^{\frac{1}{2}},$$

where *σ* is a compressive stress of the modified layer.

## 2. Review of Recent Papers on Surface Activation of Polytetrafluoroethylene (PTFE)

Polytetrafluoroethylene (PTFE) is a high-melting-point thermoplastic polymer that maintains good mechanical properties up to the melting point close to 600 K and almost perfect chemical inertness up to about 900 K, so it has been widely used in numerous applications, including nuclear power plants. The chemical inertness, however, does not allow for the proper adhesion of any additional coatings, so the modification of its wettability has been a scientific and technological challenge for decades.

**Figure 1:**
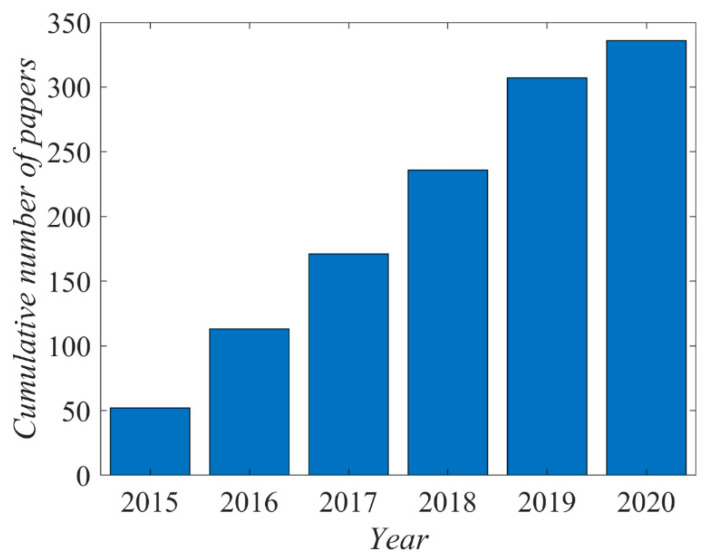
The cumulative number of scientific papers published since 2000.

The literature on the plasma activation of polytetrafluoroethylene (PTFE) by gaseous plasma treatments is vast. There are over 1300 hits when using the keywords “PTFE” and “plasma”. The cumulative number of papers is presented in Figure 1. Only the most relevant articles published since 2015 are reviewed and commented on in this paper.

The gaseous plasma treatment of PTFE may lead either to increased or decreased hydrophilicity. Carbone et al. [17] used a microwave (MW) discharge to sustain plasma at atmospheric pressure. The MW plasma jet was sustained either in pure argon or in a mixture of argon (Ar) and oxygen (O_2_). In the latter, the concentration of oxygen was fixed at 2.3 vol%. The discharge power was 50 W, and the PTFE samples were placed perpendicularly to the atmospheric pressure plasma jet. The untreated PTFE sample exhibited the water contact angle (WCA) of 110°. The WCA remained practically unchanged after the treatment in argon plasma for 15 s. Prolonged treatment for 600 s caused a decrease in the WCA to 90°. When oxygen was added, the WCA did not decrease further but instead increased to about 120°. The authors found such behavior somewhat surprising and performed X-ray photoelectron spectroscopy (XPS) to reveal any modifications of the surface composition. The surface composition, as deduced from the XPS survey spectra, showed unaltered surface composition considering the limits of the experimental error. A simple model explained the increased hydrophobicity of the samples treated with a plasma sustained in the mixture of Ar and O_2_. The authors found that atomic oxygen (or any other reactive oxygen species found in such plasma) did interact with the PTFE surface; however, the net effect of plasma treatment was etching rather than functionalization. According to Carbone et al. [17], the etching resulted in the formation of various molecules that are desorbed from the surface, including CF_2_O, CO_2,_ and CO. The authors even suggested the formation of more complex molecules with formulae C_x_F_y_O_z_. Microwave plasmas at atmospheric pressure do not provide ions of significant kinetic energy, so the increased hydrophobicity cannot be explained by the radiation damage caused by ions. The plasma used by authors represents a source of VUV radiation arising from both argon dimers and oxygen atoms. The VUV from Ar dimers causes significant bond scission as revealed recently by [18], so the somewhat increased wettability upon treatment with pure Ar plasma can be explained by this effect. The VUV radiation is suppressed significantly upon the addition of another gas (oxygen in the case of Carbone et al.), so a decreased wettability develops in the case of the Ar/O_2_ mixture.

Ryu et al. [19] also used the Ar–O_2_ mixture and found similar results as Carbone [17] except that the increased hydrophobicity was even more pronounced. They used a low-pressure gaseous plasma generated by a capacitively coupled radiofrequency discharge. The device operated at the standard industrial frequency of 13.56 MHz and had the nominal discharge power up to 600 W. The authors expected hydrophilic surface finish after treating PTFE with Ar–O_2_ plasma but found a super-hydrophobic character since the WCA increased from its original 111° to 179°. Interestingly, a slight increase in the oxygen content in the surface film probed by XPS was determined after plasma treatment. The authors also reported an almost immeasurably low sliding water contact angle, revealing a good surface finish. Upon plasma treatment, a rich surface morphology developed, as confirmed by scanning electron microscopy (SEM). The super-hydrophobic character remained almost unchanged even after 80 days. The plasma parameters of a capacitively coupled radiofrequency discharge (CCP) adopted by Ryu et al. [19] are entirely different from the ones of MW discharge used by Carbone [17]. Namely, low-pressure CCP is characterized by the significant kinetic energy of positively charged ions, as well as the extensive radiation of O atoms at about 130 nm, which is in the VUV range [12]. The positively charged ions bombard the surface of the PTFE foil, causing an extremely rich morphology with dense crown-shaped bumps of sub-micrometer lateral dimension and vertical dimension well over a micrometer. Such a rich surface morphology enabled the super-hydrophobic effect even though the surface layer, as probed by XPS, contained only about 2 at.% oxygen.

The influence of argon ion bombardment on surface morphology and thus the super-hydrophobic surface finish was further elaborated by Pachchigar et al. [20]. Instead of using gaseous plasma, they employed Ar ion beams. The ultimate pressure in the treatment chamber was as low as 10^−5^ Pa, and the working pressure 10^−2^ Pa with high-purity argon was introduced upon continuous pumping. The ion kinetic energy was adjustable between 300 and 1000 eV. SEM was used to reveal the topography. It was similar to that reported by Ryu et al. [19] except that the aspect ratio of the nanostructures was over 10. The ion radiation time was of the order of a minute. Some samples were tilted against the ion beam to obtain the topography resembling well aligned structures in the direction of the ion beam. The water contact angle increased with increasing roughness as determined by atomic force microscopy (AFM). Even half a minute of treatment was sufficient to increase the WCA from the original 105° to about 145°. The WCA increased with increasing ion energy for such a relatively short treatment time. However, at longer treatment times, the differences were found to be negligible since the water contact angle stabilized at about 150° for prolonged treatments, irrespective of Ar ion energy. The WCA also depended on the incidence angle of 300 eV ions and peaked at the incidence angle of about 40°. The effect of the incidence angle was less pronounced for 800 eV ions. Interestingly, no WCA above 150° was reported by Pachchigar et al. [20], although the surface topography was exceedingly abundant, even more than what was reported by Ryu et al. [19].

A low-pressure argon plasma sustained by capacitively coupled radiofrequency (RF) discharge was also used by Dumee et al. [21]. The discharge power was between 50 and 80 W. The gas pressure was between 20 and 80 Pa, and plasma was sustained in different gases, including argon, water vapor and air. The substrates were mesoporous membranes made from PTFE. The original WCA of such porous material was about 140°. The evolution of the WCA was determined for all three gases versus the treatment time; a well pronounced minimum appeared for all gases. The minimal contact angle for all gases was about 120°, and it appeared after a few minutes of plasma treatment. After that, the WCA increased monotonously with increasing treatment time and approached the original value typical for an untreated sample. The kinetics also depended on the discharge power and pressure. The discharge power used by Dumee et al. was roughly an order of magnitude smaller than the power used by Ryu et al. [19] what may explain the relatively meager evolution of the surface topography and thus the absence of the super-hydrophobic surface finish reported by Ryu et al. Partial de-fluorination of the surface layer of PTFE was observed by Fourier transform infrared reflectance (FTIR). Changes in wettability were directly correlated to the surface charge and zeta potential, which demonstrated that the plasma treatment was efficiently altering the surface charge of PTFE. The permeance of the membranes was found to be proportionally dependent on the surface wettability of the material.

Capacitively coupled radiofrequency discharge was also used for sustaining low-pressure oxygen plasma by Lo Porto et al. [22]. They studied the evolution of the PTFE morphology versus the treatment time at the discharge power of 200 W. Even a one minute treatment caused the formation of densely packed nanostructures of a lateral dimension of the order of 10 nm. Prolonged treatment caused the evolution of the topography. At the treatment time between 10 and 15 min, the crown-shaped bumps of about a micrometer in lateral dimension and vertical dimension well over a micrometer were observed, similar to the observations by Ryu et al. [19]. Spherical-shape objects of a micrometer diameter were observed after prolonged treatment. Unlike Ryu et al. [19], who observed only 2 at.% oxygen after plasma treatment, Lo Porto et al. [22] reported monotonously (not linear, though) increasing oxygen concentration with increasing treatment time. Unlike all other authors, Lo Porto et al. also reported the appearance of metals in the surface film as probed by XPS. Fe, Cr and Mn were found on the surface, and the concentration as deduced from the XPS survey spectra followed the concentration of oxygen. The authors provided the explanation for the evolution of the surface morphology and the formation of small spheres, which were found to consist of metal oxides rather than residues of PTFE: the powered electrode made from stainless steel was weakly sputtered by positively charged ions and the sputtered metallic atoms accumulated on the surface. As the treatment proceeded, the concentration of metal atoms becomes high enough to form the most stable geometrical form—spheres. This paper is one of the very few that report the deposition of metals upon application of capacitively coupled low-pressure RF discharge. The increased wettability of PTFE upon treatment with plasma sustained by capacitively coupled discharge is easily attributed to the deposition of metals from the powered electrode. The effect should be more pronounced when using argon as the working gas since Ar ions of kinetic energy of a few 100 eV cause significant sputtering. The effect occurs when the powered electrode (where the self-bias effect is substantial) is not covered with a polymer.

Oxygen plasma was also used as a method for the modification of PTFE high-frequency boards. Zhou et al. [23] used a powerful plasma device for the treatment of such samples with numerous holes of the sub-millimeter dimension at a low pressure of 35 Pa. A mixture of oxygen and CF_4_ was introduced into the plasma reactor, and the treatment time was about half an hour. The aim was the improved wettability of the holes and thus the better adhesion of the deposited metal. Unlike previously cited authors, Zhou et al. observed surface smoothening upon plasma treatment. The original WCA was as large as 127° but dropped to about 106° after prolonged plasma treatment. Similar to Carbone et al. [17] and Ryu et al. [19], no significant modification of the surface composition as deduced from the XPS survey spectra were observed. Although the survey spectra did not reveal measurable amounts of any other element, the broad O1s peak was deconvoluted using oxygen bonds with various elements. Implicitly, this report indicates the sputtering of the electrode material. As a result, the adhesion of copper on the plasma-treated PTFE improved. The best results were reported using a gas mixture of 200 sccm of CF_4_ and 300 sccm of O_2_. The lengthy treatment time (half an hour) indicates the low etching of the electrode material by positively charged ions. Namely, the sputtering coefficient for Ar^+^ ions is higher than for positively charged ions formed in plasma sustained in CF_4_/O_2_ mixtures.

A similar result was reported by Mi et al. [24], who studied the influence of oxygen plasma treatment of PTFE on surface properties, specifically biological response. A capacitively coupled RF discharge at the power of 200 W was used to sustain plasma at low pressure in pure oxygen. The WCA dropped to about 90° after half an hour of plasma treatment. Although the wettability of plasma treatment samples was not great, the high resolution XPS C1s peak showed a dramatic increase in the C–C bond at the binding energy of about 285 eV at the expense of the C–F_2_ bond at about 292 eV. Hydroxyl groups were also found in the high-resolution C1s peak after the plasma treatment. Much less apparent modifications were observed by FTIR. SEM images did not reveal a dramatic change in the topography, although the treatment time was similar to that by Ryu et al. [19]. The plasma reactors adopted by Mi et al. [24], Dumee et al. [21] and Ryu et al. [19] are similar—they all operate at low pressure using capacitively coupled RF discharges. The resultant surface finishes, however, are entirely different, indicating the importance of details. Here, it is worth mentioning that the treatment time adopted by Mi et al. [24] falls into the saturation regime observed by Dumee et al. [21].

The moderately hydrophilic character of the PTFE substrates with water contact angles of about 50° was observed by Valerio et al. [25]. The original WCA was 118°. They used a microwave discharge at low-pressure conditions (150 Pa) and discharged power of 700 W. Ar was used as working gas, but the ultimate vacuum was poor; hence, a quite large concentration of water vapor persisted upon sustaining argon plasma. The useful treatment time was up to one minute. FTIR revealed the formation of an OH stretching vibration band at 2920 cm^−1^ after plasma treatment. The deconvolution of the XPS C1s band revealed both hydroxyl and other functional groups. No hydrophobic recovery was observed for up to a week of aging at ambient conditions. Unlike Carbone et al. [17], who also used an MW discharge, the plasma sustained by Valerio et al. [25] was rich in OH, H and O radicals since it was contained in a glass chamber at low pressure. The molecules or residual atmosphere (predominantly water vapor) were effectively dissociated upon the electron-impact dissociation and interaction with Ar metastables. The loss rate for radicals such as OH, H and O on a glass surface is relatively low [26,27], so the density is high. In addition, the OH radicals are extensively excited at low pressure, causing the strong emittance of UV radiation with the bandhead at about 309 nm. The atomic species in low-pressure plasma radiate also in the VUV range. The improved wettability, as observed by Valerio et al., may be explained by the combined effect of plasma radicals and UV/VUV radiation. Unfortunately, the concentration of water vapor in the system was not reported. Since the experiments were performed in a simplified reactor that was never baked and pumped only by a simple rotary pump, the water vapor partial pressure can be estimated to 1–2 Pa.

The moderately hydrophilic character of PTFE after plasma treatments was also reported by Karoly et al. [28]; however, they used a completely different discharge than previously cited authors. Plasma was sustained by a high-impedance coplanar atmospheric pressure discharge operating in air at the frequency of 10–20 kHz with a 20 kV peak-to-peak voltage and a power of 320 W. The treatment times were between 30 and 600 s. The original WCA, which was 108°, slowly decreased with increasing treatment time and dropped to about 65° after 600 s of treatment. A slow hydrophobic recovery was observed over several weeks of storage at ambient conditions. XPS survey spectra revealed a significant de-fluorination of the surface film since the F/C ratio dropped from 2 to 0.9. The oxygen and nitrogen concentrations in the surface film probed by XPS were 3 at.% and 2 at.%, respectively. Interestingly, the increased wettability did not result in the better adhesion of various glues. The positive effect of the plasma treatment, as reported by Karoly et al. [28], was a significant decrease in the standard deviation of the adhesive forces.

A similar discharge was also used by Krumpolec et al. [29]. Unlike other authors who pre-cleaned the PTFE samples by chemical methods before plasma treatment, they used as-received samples. As a consequence, a thin film of hydrocarbons was found by XPS even for an untreated sample. The F/C ratio for the untreated sample was only 1 (for the PTFE of the contaminant-free surface should be 2). Even 2 s of plasma treatment caused a significant increase in the F/C ratio indicating the rapid removal of the organic pollutant from the surface. The complex behavior of the concentration of C, F and O in the surface film as probed by XPS was reported. Still, finally, after about a minute of plasma treatment, the ratio stabilized at about 1.8. The deviation from the theoretical value was explained by the partial substitution of F from PTFE with O. Although Krumpolec et al. used almost identical conditions as Karoly et al. [28], no nitrogen was observed on the surface of PTFE after plasma treatment. Both authors used XPS for probing the surface composition. Krumpolec et al. did not report the water contact angles, but they reported excellent surface finish, which enabled the deposition of Al_2_O_3_ thin films. The results reported by Krumpolec et al. [29] reveal the importance of organic surface contaminants on the wettability as well as the fact that the atmospheric pressure discharge in this mode does not enable the fast removal of the contaminants.

The adhesion properties were also studied by Ohkubo et al. [30]. They used atmospheric pressure plasma in helium gas of commercial purity of 99.99%. Plasma was sustained by a capacitively coupled RF discharge at the power density of 7.4 W/cm^2^. Samples were additionally heated up to about 200 °C upon plasma treatment. Unlike previously cited authors, a large concentration of oxygen (32 at.% and 35 at.% for treatment times of 50 and 600 s, respectively) was found by XPS after the plasma treatment. However, no oxygen-containing gas was introduced intentionally into the discharge. The original WCA was 115°, and it dropped to 43° after 50 s plasma treatment at the temperature of 95 °C. The water contact angle was 109° after treatment with the plasma of the same duration but at the temperature of 205 °C. Surprisingly enough, the best adhesion for epoxy resin was observed after the treatment at 205 °C, where the contact angle measurements revealed a moderately hydrophobic surface finish, although the oxygen content was still 25 at.%. The results provided by Ohkubo et al., therefore, indicate a complex relationship between wettability, oxygen concentration and adhesion. The large surface concentration of oxygen, as determined by XPS, may be explained by extensive VUV radiation arising from He dimers [18].

Plasma sustained in helium at atmospheric pressure was also used for the modification of PTFE powder. Yoo et al. [31] adopted a high-impedance surface dielectric barrier discharge to sustain the plasma in a gap between the electrodes using a 30 kHz power supply of voltage about 1 kV peak-to-peak. The discharge appeared in pulses of a typical duration of about 1.5 µs. A small admixture of H_2_ and NF_3_ was used to obtain the desired surface finish of PTFE powder. The authors claim that the addition of hydrogen enabled the creation of dangling bonds and breaking C–F bonds in the surface layer. High-resolution XPS C1s peaks revealed a significant depletion of fluorine from the surface film probed by XPS, but practically no oxidation occurred. Still, the plasma treatment enabled the reasonable suspension of sub-micrometer PTFE dust particles in aqueous solutions.

Gliding arc is another discharge suitable for sustaining moderately non-equilibrium gaseous plasma at atmospheric pressure. Hong et al. [32] performed a treatment of PTFE films using different gases. The discharge was powered at a frequency of 25 kHz. The treatment times were up to 30 min long. The original WCA (for untreated PTFE) was as low as 80°. The contact angle decreased monotonously with the increasing treatment time when the gliding arc was sustained in oxygen. For prolonged treatment, the lowest WCA of 45° was observed for oxygen plasma, followed by argon (49°), nitrogen (51°), and air (61°). No correlation between the WCA and the oxygen concentration, as probed by XPS, was observed. In fact, by far the largest oxygen concentration was found in the surface film of the samples treated with argon plasma—as large as 19 at.%. For the oxygen plasma treatment, the concentration was solely 3 at.%. Somewhat unexpected results were explained by the chemisorption of oxygen after accomplishing argon plasma treatment. As mentioned earlier, argon plasma sustained at atmospheric pressure is a rich source of VUV radiation, which breaks bonds between C and F atoms and is beneficial for wettability.

The wettability of PTFE can also be tailored upon the sputter-deposition of the thin polymer films resembling PTFE. Prysiazhnyi et al. [33] performed sputtering of the PTFE substrate using magnetized capacitively coupled RF plasma of discharge power 50 W and a frequency of 13.56 MHz operating at the pressure of 1.5 Pa. The treatment time was half an hour. Despite the low base pressure of 5 × 10^−4^ Pa, the optical emission spectroscopy revealed an OH band between 305 and 320 nm. When pure argon was used, the oxygen concentration in the deposited film, as probed by XPS, was about 1 at.% and the water contact angle around 100°. Mixing argon with nitrogen at 50:50 caused the deposition of a PTFE-like film of about 6 at.% nitrogen, but the water contact angle remained practically unchanged. The authors found that the addition of acetylene into the gas mixture upon sputter deposition beneficial for increased wettability. The WCA dropped to about 17° in a broad range of C_2_H_2_ concentrations spanning from about 10 vol.% to 25 vol.%. Interestingly, a large concentration of oxygen (11–19 at.%, depending on gas mixture) was found by XPS despite the fact that no oxygen was added into the gas mixture upon deposition of PTFE-like films at low pressure. Since the ultimate pressure was low, it was assumed that the surface oxidation occurred upon the exposure of freshly prepared polymer films to the ambient atmosphere.

The best results in terms of the hydrophilic surface finish were reported recently by Nguyen et al. [34]. The WCA dropped from the original 118° down to 4° only. They used an inductively coupled RF discharge at the absorbed power of about 100 W. Several gases were tested. The treatments with oxygen and argon plasmas did not show significant modifications since the WCA remained at 112° and 110° after both treatments, respectively. Using plasma sustained in argon, ammonia and water, however, caused fluorine elimination from the surface film and hydrophilic functional groups containing oxygen and nitrogen were found on the PTFE surface. Roughening the surface was observed to coincide. The rich morphology and functionalization with polar groups caused the super-hydrophilic effect, which was observed after about 15 min of plasma treatment. The F/C ratio, as probed by XPS, dropped from the original 2.07 to merely 0.31, and the O/C ratio after the treatment was about 0.2. A significant concentration of nitrogen was reported as well. Samples were always kept at floating potential during the plasma treatment, so the kinetic energy of ions was negligible. Plasma sustained by inductively coupled discharges in glass tubes was characterized by the extremely high dissociation of gaseous molecules [35]. The flux of H, N and NH radicals on the surface in the reactor adopted by Nguyen et al. [34] is, therefore, considerable. Furthermore, such plasma is a tremendous source of radiation [36], so the combined effect of UV/VUV radiation causes the rapid depletion of fluorine and substitution with NH_x_ and OH groups. The super hydrophilic effect probably occurs in a limited range of gas concentrations. Nguyen et al. reported the best results at the Ar flow of 64 sccm and NH_3_ and H_2_O flow rates of 0.43 mmol/min and 0.04 mmol/min, respectively [34].

## 3. Correlations between Treatment Parameters and Surface Wettability

Different authors have used different discharges useful for the surface modification of polytetrafluoroethylene samples, so the results are difficult to compare. Still, it is often helpful to draw correlations between the processing parameters and the surface finish. Table 1 represents the key observations provided by the above cited documents. The data presented in Table 1 enable drawing correlations between the treatment parameters and surface finish. The relationships are dim for almost all parameters, but some correlations could be drawn nonetheless.

**Figure 2:**
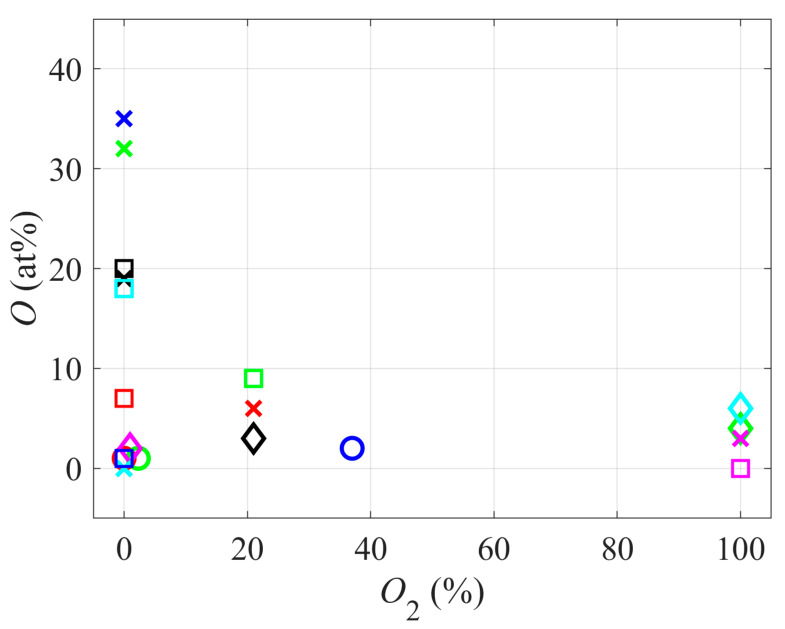
The concentration of oxygen in the surface film versus the concentration of oxygen in gaseous plasma.

Figure 2 represents the correlation between the concentration of oxygen in the surface film as probed by XPS and the concentration of oxygen in the gas used for sustaining plasma. Interestingly enough, the concentration of oxygen in the polymer surface decreases with the increasing concentration of oxygen in the gaseous plasma. The highest concentration of oxygen in the polymer surface film is observed for plasmas where no oxygen was added (at least not intentionally), or the oxygen concentration in the gas phase was marginal (well below 1 vol.%). This observation is specific for the plasma activation of PTFE. Notably, for most fluorine-free polymers, the hydrophilic surface finish is usually achieved by a brief treatment with a plasma sustained in pure oxygen or a mixture of oxygen with a noble gas. The discrepancy is explained by different compositions between PTFE and other polymers. Each carbon atom in the PTFE polymer chain is bonded to two F atoms. The C–F bond is among the highest of all chemical bonds, so the bond cleavage requires high energy. Furthermore, fluorine has the highest oxidation potential among all elements; hence the substitution of F-atoms with O or OH radicals is thermodynamically unfavorable. The interaction of reactive oxygen species from gaseous plasma, therefore, does not lead to the substitution of F in the surface film with O. The energy dissipated upon the interaction with energetic oxygen species (in particular O_2_^+^ ions impinging the PTFE surface) is sufficient for breaking bonds. Still, the predominant reaction is etching rather than functionalization with oxygen. As disclosed by Carbone et al. [17], the interaction between reactive oxygen species and the PTFE surface leads to the formation of volatile C_x_F_y_O_z_ molecules, which desorb from the surface; therefore, the net effect is etching.

**Figure 3:**
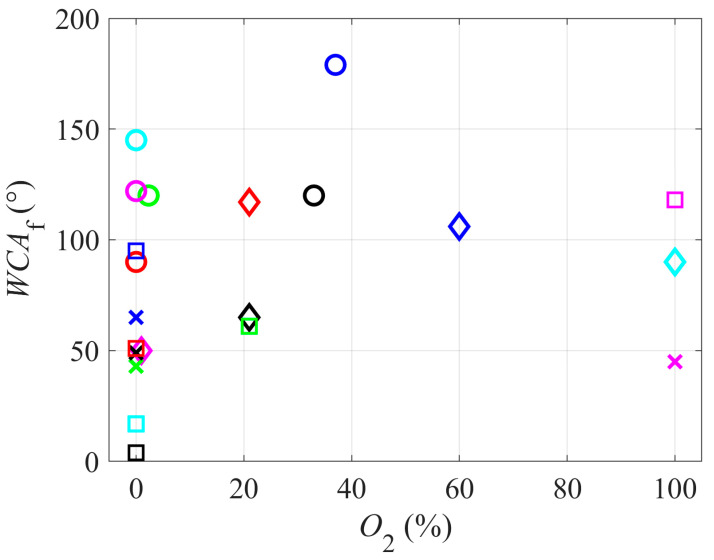
Water contact angle versus the concentration of oxygen in the gaseous plasma.

Figure 3 represents the correlation between the water contact angle and the concentration of oxygen in the gaseous plasma. A variety of WCAs has been reported with the absence of oxygen in plasma (or at least no intentional addition of an oxygen-containing gas into the gas mixture), ranging from a super-hydrophilic surface finish (very small WCA) to highly hydrophobic finish (WCA close to 150°). In such cases, the wettability is apparently governed by little details. Such details include the ion kinetic energy and the intensity of UV/VUV radiation. Unfortunately, very few authors reported the energy of positively charged ions impinging the surface and no author cited in this review reported the flux of radiation, so such correlations cannot be presented.

**Figure 4:**
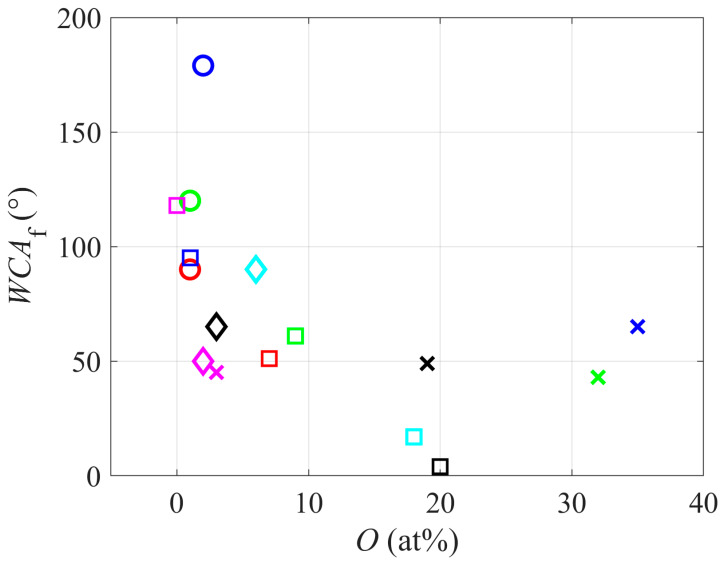
Water contact angle versus the concentration of oxygen in the polymer surface film.

Intriguing is the correlation between the water contact angle and the concentration of oxygen in the surface film as probed by XPS. The results reported by all authors are plotted in Figure 4. The correlation was as expected: a higher concentration of oxygen in the surface film reflects a lower WCA, thus better wettability. The data in Figure 4 are scattered, but a very low concentration of oxygen always causes high WCAs. Once the oxygen concentration in the surface film reaches almost 10%, the water contact angle remains at about 50°. Such a contact angle reveals moderate wettability. The super-hydrophilic finish was only reported in one paper. Such a surface finish is a result of two effects: surface functionalization with polar functional groups (usually oxygen groups) and rich morphology at the sub-micrometer scale. Here, it is worth mentioning that XPS probes the surface film of a thickness of several nanometers. The integral concentration of oxygen, as revealed by XPS, does not necessarily reflect the concentration of polar groups on the very surface of the polymer samples. Furthermore, the interpretation of XPS spectra taken on rough surfaces might not be trivial. Still, the correlation in Figure 4 is obvious, so the general rule for all polymers is also applicable for PTFE.

**Figure 5:**
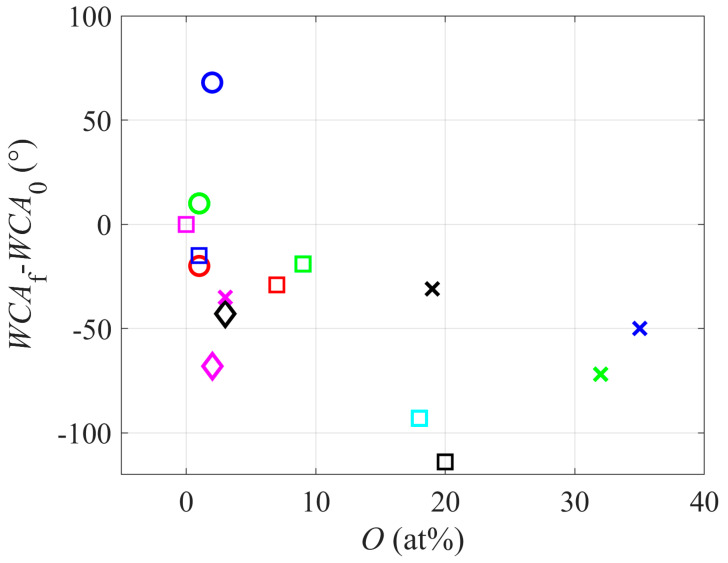
Change in the water contact angle versus oxygen concentration in the polymer surface film.

The initial WCAs (for untreated samples), as reported by different authors, are also presented in Table 1. The values are scattered between about 80° and 140°, depending on the morphology and cleanliness of the virgin samples. An interesting correlation is a change in the water contact angle, i.e., the original WCA subtracted from the final WCA. This correlation is plotted in Figure 5. As expected, most authors reported a significant drop in the water contact angle. Some, however, found a marginal drop or even an increase in the water contact angle. For example, Ryu et al. [19] reported the final WCA as high as 179° (the initial WCA was 111°). The observation was explained by the extremely rich morphology obtained due to PTFE treatment with a plasma sustained in a mixture of Ar and 37 vol.% O_2_. Namely, SEM images revealed the formation of crown-shaped bumps. It is known that the super-hydrophobic effect is a result of non-polar surface groups and rich morphology on the sub-micrometer scale [37].

**Figure 6:**
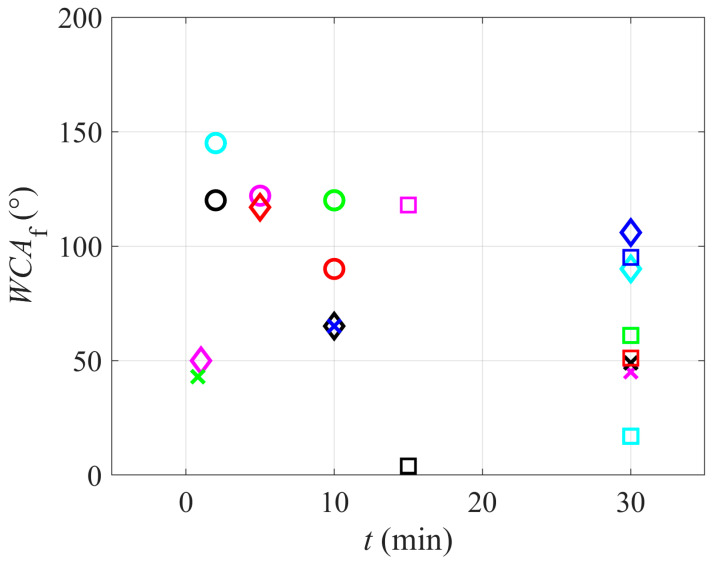
Water contact angle versus the treatment time.

The relationship between the treatment time and wettability is worth discussing. Figure 6 is a plot of the reported WCAs versus the treatment time. One can hardly observe any correlation. The diagram in Figure 6, therefore, reveals that the treatment time is not the factor that influences the PTFE wettability, so one can conclude that some sort of saturation effects occur versus the treatment time. Unfortunately, very few authors reported the temporal evolution of the surface wettability, so the results may not be statistically significant.

## 4. Interaction Mechanisms

**Figure 7:**
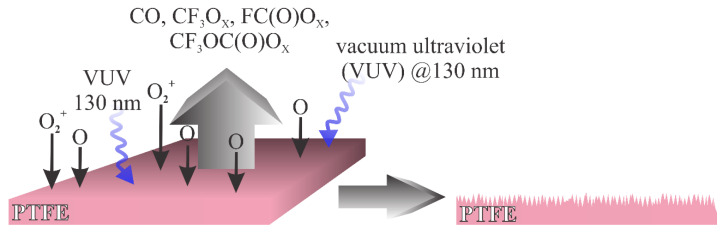
Schematic of the interaction between oxygen plasma and PTFE.

Based on the above review, it is possible to distinguish between two types of plasma treatments that enable a different surface finish: (i) treatment with plasma rich in reactive oxygen species, and (ii) treatment with plasma that contains a minute quantity of oxygen species but is rich in reactants that cause bond-scission and thus the depletion of fluorine from the surface film. The first one is illustrated in Figure 7. In cases where the gaseous plasma is rich in oxygen, the radiation that causes bond breaking between C and F atoms in PTFE arises practically only from excited neutral oxygen atoms. The radiation appears at the wavelength around 130 nm [12]. The radiation is not extensive enough to cause significant bond breakage, but the reactive oxygen species readily interact chemically with the PTFE surface causing oxidation. The result is a formation of unstable fragments containing carbon, fluorine and oxygen. Known fragments of such type include oxy (x = 1) and peroxy (x = 2) radicals of formulae CF_3_O_x_, FC(O)O_x_, CF_3_C(O)O_x_ and CF_3_OC(O)O_x_. Such moieties desorb from the surface, which results in etching. The etching outcome is increased roughness, which in turn results in the super-hydrophobic surface finish. A typical example of such a mechanism was elaborated by Ryu et al. [19].

**Figure 8:**
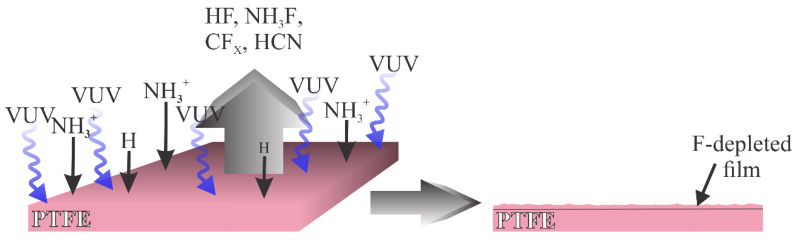
Schematic of the interaction between plasma rich in VUV radiation and PTFE.

Another type of plasma used in a modification of PTFE is plasma rich in VUV radiation. Such plasma was used by Nguyen et al. [34], and more recently by Lojen et al. [38]. The interaction of such plasma with the PTFE surface is illustrated in Figure 8. Here, the flux of oxygen plasma species is marginal compared to the flux of the radiation arising from hydrogen molecules and atoms. The PTFE surface is thus exposed to the VUV radiation that causes the C–F bond scission. Simultaneously, the PTFE surface is exposed to radicals that may interact with F atoms on the surface, for example, NH_3_^+^, as proposed by Nguyen et al. [34], and H atoms as elaborated by Lojen et al. [38]. The PTFE surface layer is thus depleted of fluorine, and the oxidation of the dangling bonds occurs by interaction with OH radicals that are likely to be presented in a low-pressure plasma reactor in minute quantities. An alternative may be surface oxidation upon venting the plasma reactor. Namely, the oxidation of polymer samples treated by VUV radiation in the absence of plasma conditions occurs spontaneously as the sample is exposed to ambient conditions [39].

## 5. Conclusions and Roadmap

The review of recent results of the plasma treatment of polytetrafluoroethylene indicates that the mechanisms governing the surface finish are still far from being well understood. At least a moderate concentration of oxygen functional groups on the PTFE surface is essential for improved wettability, the same as for other polymers. A trivial solution for the activation of PTFE materials is the deposition of a foreign material onto the polymer surface. The deposition is achievable by using gaseous plasma sustained with a discharge that enables the high kinetic energy of ions impinging an electrode. Widely used is capacitively coupled high-frequency (possibly RF) discharge. The smaller electrode of such a discharge is self biased to a relatively high negative potential that attracts positively charged ions from gaseous plasma. The ions cause sputtering of the electrode material and thus, the deposition of a material with high surface energy. The sputtering is most efficient when the sheath between the plasma and electrode is almost collisionless, therefore at low pressure. Such a solution, however, does not enable the good adhesion of any coating since there is a weak chemical interaction between PTFE and the deposited material.

A non-trivial method for achieving the good wettability of PTFE is treatment with a gaseous plasma sustained in a gas mixture with a minimal content of oxygen or oxygen-containing gas. Radiation in the invisible range (UV and in particular VUV) causes bond scission and thus, fluorine depletion on the polymer surface. The radiation depends enormously on the type of discharge and gaseous impurities. Especially impurities tend to quench metastables and cause a decrease in the electron temperature, so the radiation in the deep UV range is suppressed. Furthermore, the VUV radiation is intensively absorbed in ambient gas, so it is better to place the polymer samples close to the source of radiation, i.e., in the region of most luminous plasma. Both noble and some molecular gases are rich sources of VUV radiation arising from transitions of highly-excited atoms or molecules. At atmospheric pressure, the most intensive radiation occurs from excited Ar or He dimers, whereas at low pressures, the radiation from atoms is often as intensive, if not more so.

The massive scattering of results reported by different authors suggests that the processing parameters such as the treatment time, type of gas, discharge power, or frequency are not the most appropriate for studying the evolution of the surface wettability or surface functionalization with oxygen functional groups. A better choice of parameters would include fluences (or fluxes) of reactive gaseous species and invisible radiation. Such parameters are not trivial to determine, so the complete characterization of gaseous plasma represents a scientific challenge that will enable understanding the complex surface mechanisms involved upon the plasma treatment of polytetrafluoroethylene.

The VUV radiation is rarely measured upon the treatment of polymers with gaseous plasma. Since the ratio between VUV and reactive oxygen species seems to be the decisive factor for the surface wettability, a trend in the modification of PTFE polymer materials in plasma conditions is the estimation of the fluxes and/or fluences of such radiation, for example, using a technique as elaborated by Fanz et al. [36]. The desired surface finish is obtained by a proper choice of the type of plasma radicals and fluences. From a scientific point of view, the best method for explaining the surface finish would be by adjusting the fluxes of VUV radiation and chemically reactive plasma species independently, as demonstrated recently by Lojen et al. [38].

## Figures and Tables

**Table 1:** Summary of treatment parameters and surface finish of polytetrafluoroethylene (PTFE) reported by different authors.

Reference	Gas	O_2_ (%) ^1^	O (at.%) ^2^	WCA_0_ (°) ^3^	WCA_f_ (°) ^4^	P (W) ^5^	p (mbar) ^6^	f (MHz) ^7^	t (min) ^8^
Carbone et al. [17]	Ar	0	1	110	90	50	1000	2450	10
Carbone et al. [17]	Ar	2.3	1	110	120	50	1000	2450	10
Ryu et al. [19]	Ar	37	2	111	179	150	0.13	13.56	180
Pachchigar et al. [20]	Ar	0	N/A ^9^	105	145	N/A	0.01	0	2
Dumee et al. [21]	Ar	0	N/A	140	122	80	0.6	13.56	5
Dumee et al. [21]	H_2_O	33	N/A	140	120	80	0.2	13.56	2
Dumee et al. [21]	air	21	N/A	140	117	80	0.2	13.56	5
Lo Porto et al. [22]	O_2_	100	4	N/A	N/A	200	0.13	13.56	1
Zhou et al. [23]	CF_4_	60	N/A	127	106	8000	0.35	N/A	30
Mi et al. [24]	O_2_	100	6	N/A	90	200	0.1	N/A	30
Valerio et al. [25]	Ar	1	2	118	50	700	1.5	2,450	1
Karoly et al. [28]	Air	21	3	108	65	320	1000	0.015	10
Krumpolec et al. [29]	Air	21	6	N/A	N/A	320	1000	0.015	1
Ohkubo et al. [30]	He	0.0001	32	115	43	7.4	1000	13.56	0.8
Ohkubo et al. [30]	He	0.0001	35	115	65	7.4	1000	13.56	10
Yoo et al. [31]	He	0.0001	0	N/A	N/A	N/A	1000	0.03	30
Hong et al. [32]	O_2_	100	3	80	45	N/A	1000	0.025	30
Hong et al. [32]	Ar	0	19	80	49	N/A	1000	0.025	30
Hong et al. [32]	N_2_	0	7	80	51	N/A	1000	0.025	30
Hong et al. [32]	Air	21	9	80	61	N/A	1000	0.025	30
Prysiazhnyi et al. [33]	Ar	0.0001	1	110	95	50	0.015	13.56	30
Prysiazhnyi et al. [33]	Ar + N_2_ + C_2_H_2_	0.0001	18	110	17	50	0.015	13.56	30
Nguyen et al. [34]	O_2_	100	0	118	118	100	N/A	13.56	15
Nguyen et al. [34]	Ar + NH_3_ + H_2_O	0.002	20	118	4	100	N/A	13.56	15

^1^ O_2_ (%) indicates the concentration of oxygen in the gaseous plasma. ^2^ O (at.%) indicates the concentration of oxygen in the surface film of PTFE as probed by XPS. ^3^ WCA_0_ (°) indicates the water contact angle of the untreated samples. ^4^ WCA_f_ (°) indicates the final water contact angle determined just after plasma treatment. ^5^ P (W) indicates the reported discharge power. ^6^ p (mbar) indicates the total gas pressure upon plasma treatment.^7^ f (MHz) indicates the frequency of the power supply. ^8^ t (min) indicates the treatment time. ^9^ N/A indicates no data could be deduced from the reports.
